# Associations of AI-derived coronary CT angiography features with CZT-SPECT coronary flow reserve and FFR-CT

**DOI:** 10.3389/fcvm.2026.1803774

**Published:** 2026-04-21

**Authors:** Jing Ni, Zekun Pang, Haoran Guo, Ajay Kumar Chaudhary, Fukai Zhao, Yue Chen, Jiao Wang, Jianming Li

**Affiliations:** 1 Clinical College of Cardiovascular Diseases, Tianjin Medical University, Tianjin, China; 2 Department of Nuclear Medicine, TEDA International Cardiovascular Hospital, Tianjin, China

**Keywords:** artificial intelligence, cadmium-zinc-telluride single-photon emission computed tomography, coronary computed tomography angiography, coronary flow reserve, fractional flow reserve derived from coronary CT angiography

## Abstract

**Objective:**

This study aims to explore the associations of artificial intelligence (AI)-derived coronary CT angiography (CCTA) features with coronary flow reserve (CFR) measured by cardiac-cadmium zinc-telluride single-photon emission computed tomography (CZT-SPECT) and CT-derived fractional flow reserve (FFR-CT), and to investigate their intrinsic relationships.

**Methods:**

This retrospective study included 251 patients (753 vessels) with suspected or known coronary artery disease (CAD), who underwent CZT-SPECT and concurrent CCTA. Myocardial ischemia was defined as CFR <2.0 or FFR-CT ≤0.8. Generalized estimating equations (GEE) were used to analyze the associations between CCTA coronary parameters and the two ischemia definitions.

**Results:**

Among the 753 vessels, the agreement analysis between CFR and FFR-CT for ischemia was poor (Kappa = 0.084). Multivariate analysis demonstrated that CFR <2.0 was only associated with perivascular fat attenuation index (FAI) and calcified plaque burden, whereas FFR-CT ≤0.8 was additionally predicted by low attenuation plaque and lipid plaque burden (all *p* < 0.05). Subgroup analysis revealed distinct plaque feature patterns among discordant CFR/FFR-CT statuses. The same set of coronary features achieved an adjusted AUC of 0.892 for FFR-CT-defined ischemia and 0.615 for CFR-defined ischemia.

**Conclusions:**

CFR and FFR-CT reflect different pathophysiological dimensions: CFR reduction is more associated with microvascular dysfunction in the context of inflammation and diffuse lesions, whereas FFR-CT mainly reflects focal, obstructive ischemia caused by high-risk plaques (such as low attenuation plaque). CCTA is an important tool for assessing obstructive coronary lesions, but coronary features alone are insufficient to predict whether CFR is abnormal or not. However, in the absence of invasive reference standards (invasive FFR and index of microcirculatory resistance), these findings should be considered hypothesis-generating and require confirmation in future studies incorporating invasive physiological assessment.

## 1 Introduction

Coronary artery disease (CAD) is currently the leading single cause of death worldwide, responsible for nearly 7 million deaths annually and imposing a substantial global economic and social burden (1). Its main pathophysiological mechanism is myocardial ischemia, which can be caused by obstructive coronary atherosclerotic plaques and/or microvascular dysfunction, ultimately leading to clinical symptoms (such as chest pain), reduced quality of life, myocardial injury, and major adverse cardiovascular events (MACE) (2, 3). Therefore, the precise diagnosis of CAD requires attention to both coronary morphological changes and the assessment of myocardial ischemia. In recent years, with the advancement of medical imaging technologies, radionuclide imaging-derived coronary flow reserve (CFR) and fractional flow reserve derived from coronary CT angiography (FFR-CT) have become important non-invasive functional quantitative indicators for evaluating myocardial ischemia in CAD. Previous studies have demonstrated their good correlation with invasive methods, and their clinical value is widely recognized (4–7). Among these, cardiac-dedicated cadmium-zinc-telluride single-photon emission computed tomography (CZT-SPECT) enables non-invasive measurement of coronary flow reserve (CFR), showing strong agreement with results from positron emission tomography (PET), and its clinical application is increasing (8–10). Meanwhile, FFR-CT and artificial intelligence (AI)-based parameters derived from coronary computed tomography angiography (CCTA) are also advancing rapidly in both technological development and clinical implementation (11). Thus, the era of multi-modality imaging for the diagnosis and assessments of CAD has arrived (12). However, in clinical practice, discrepancies between CFR and FFR-CT results are often encountered. Although previous studies have elucidated their respective mechanisms: CFR reflects the overall coronary circulation reserve (including epicardial and microvascular components) (13), whereas FFR-CT indicates the physiological significance of flow-limiting coronary stenosis (14). There remains a lack of research on the relationship and differences between CFR and FFR-CT in evaluating myocardial ischemia in CAD, as well as on the predictive and diagnostic performance of AI-derived CCTA-based coronary features. Therefore, this study aims to explore the associations of AI-derived CCTA features with CFR and FFR-CT, and to explore their intrinsic relationships.

## 2 Materials and methods

### 2.1 Study design

**Figure 1:**
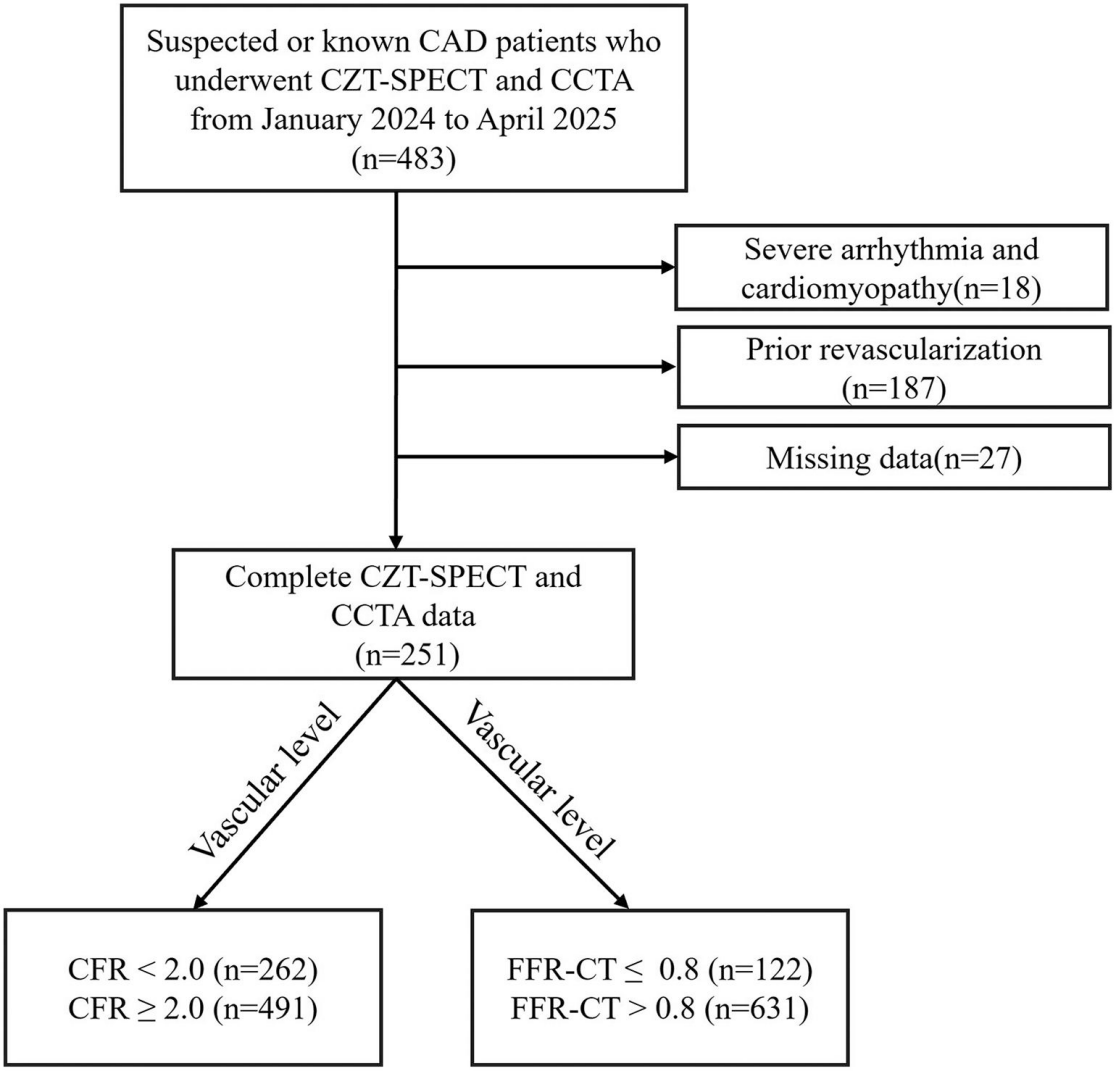
The screening schematic diagram of this study. CAD, coronary artery disease; CCTA, coronary computed tomography angiography; CZT-SPECT, cadmium-zinc-telluride single-photon emission computed tomography; CFR, coronary flow reserve; FFR-CT, fractional flow reserve derived from coronary CT angiography.

Clinical and imaging data were retrospectively collected from patients with suspected or known CAD who underwent both cardiac-dedicated CZT-SPECT dynamic myocardial perfusion imaging (DMPI) and CCTA at our hospital from January 2024 to April 2025. Inclusion criteria were: age ≥18 years; interval between CCTA and CZT-SPECT < 3 months, with no revascularization during this period; complete clinical and imaging data. Exclusion criteria included: prior revascularization; history of heart failure or myocardial infarction; severe arrhythmia or cardiomyopathy; poor image quality or missing data. The screening process is shown in Figure 1. Finally, a total of 251 patients (753 major epicardial coronary arteries) were included. CFR <2.0 on CZT-SPECT was defined as impaired flow reserve (CFR+), and CFR ≥2.0 as normal (CFR-) (15). FFR-CT ≤0.80 derived from AI-assisted CCTA was defined as hemodynamically significant stenosis (FFR-CT+), and FFR-CT >0.80 as normal (FFR-CT−) (14). Accordingly, all vessels were categorized into four subgroups: CFR+/FFR-CT−, CFR-/FFR-CT+, CFR+/FFR-CT+, and CFR-/FFR-CT−. Obstructive CAD was defined as ≥50% diameter stenosis in any major epicardial vessel as assessed by CCTA. All participants provided written informed consent prior to the examination. The study adhered to the Helsinki Declaration and was approved by our hospital ethics committee (Ethics Number: 2026-0109-3).

### 2.2 Imaging method

#### 2.2.1 CCTA

**Figure 2:**
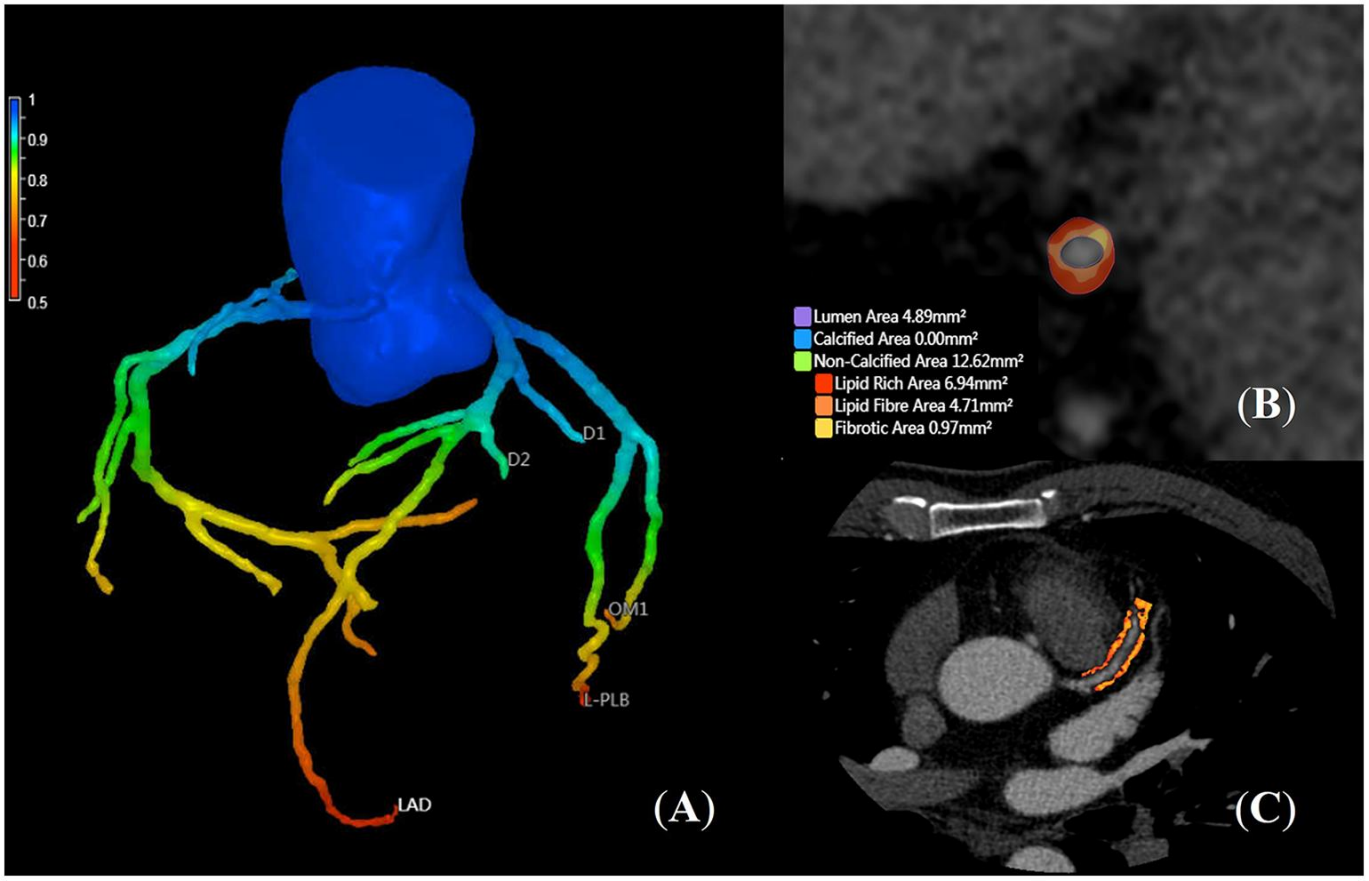
Quantitative analysis of CCTA coronary characteristic parameters. A 49-year-old male patient was admitted due to intermittent chest tightness and chest pain for 3 years, worsening over the past 10 days. CZT-SPECT showed CFR values of 1.87 in the LAD, 1.41 in the LCX, and 1.64 in the RCA. **(A)**: Computational fluid dynamics simulation based on CCTA yielded FFR-CT values of 0.77 in the LAD, 0.91 in the LCX, and 0.89 in the RCA. **(B)**: Quantitative AI analysis of the mid-LAD plaque revealed a non-calcified plaque rich in lipid content, with two high-risk features: positive remodeling and low attenuation plaque. **(C)**: AI-automated measurement showed a FAI value of −79.21 HU around the LAD.

Imaging was performed using a GE 256-slice CT scanner (Revolution, GE Healthcare, USA) with prospective ECG-gating. Ioversol contrast (350 mgI/mL, 60–70 mL) was injected at 4.5–5 mL/s. Scanning parameters were 100 or 120 kV and Smart mA (300–740 mA) (16). Data were analyzed using an AI-assisted software (uFFR-CT version 1.5, United-Imaging Healthcare, Shanghai, China). Plaque segmentation and centerline extraction were manually corrected when necessary. Typical cases are demonstrated in Figure 2. Plaque features and FFR-CT values were recorded for the left anterior descending (LAD), left circumflex (LCX), and right coronary (RCA) arteries. Statistical parameters included (17, 18): (a) Coronary artery calcium scores (CACS), defined using the Agatston calcification score in Agatston units (AU), with severity levels 0 (normal), 1–99 (mild), 100–400 (moderate), and > 400 (severe). (b) Perivascular fat attenuation index (FAI), defined as the mean CT attenuation (−190 to −30 HU) of perivascular adipose tissue. For the LAD and LCX, the proximal 40 mm segment was analyzed; for the RCA, proximal 10 mm excluded, analyzed at 10–50 mm to avoid aortic wall interference. The FAI threshold was −70.1 HU. (c) High-risk plaque features: positive remodeling (remodeling index ≥1.1); low attenuation plaque (mean CT density <30 HU); napkin-ring sign (ring-like peripheral enhancement around a hypodense core); spotty calcification (<3 mm length and peak density > 130 HU). (d) Plaque burden and volume by type: calcified (>350 HU), lipid (−30 to 30 HU), fibrous fatty (30 to 130 HU), and fibrous plaque (130 to 350 HU).

#### 2.2.2 CZT-SPECT DMPI

CZT-SPECT (NM530c, GE Healthcare, Haifa, Israel) imaging was performed using ^99m^Tc-MIBI or ^99m^Tc-TF. Patients were instructed to abstain from caffeine/theophylline-containing products for 24 h, withhold cardiovascular medications (e.g., beta-blockers, calcium channel blockers, vasodilators) for 24 h, and refrain from smoking on the day of the examination (19). Imaging was performed using a same-day protocol with rest followed by adenosine stress acquisition. Dynamic list-mode data were processed on the MyoFlowQ workstation (Beijing Larkcloud Biomedical, Beijing, China) for quantitative myocardial blood flow analysis. For detailed imaging and post-processing methods, please refer to our published literature (20). Finally, CFR values were obtained for the LAD, LCX, and RCA perfusion territories, respectively.

### 2.3 Statistical analysis

Statistical analysis was performed using SPSS 27.0 (IBM Corp., Armonk, NY, USA) and R software version 4.5.1 (R Foundation for Statistical Computing, Vienna, Austria) with the packages corrplot, pROC, boot, rms, and ggplot2. Continuous variables, presented as median (interquartile range) due to non-normal distribution, and categorical variables, presented as frequency (percentage), were analyzed for correlations using Spearman's rank test. From the initial set of CCTA parameters, representative coronary parameters were selected based on a combined approach that considered: correlation assessment (variables with correlation coefficient >0.7 were carefully evaluated for redundancy); univariate association with outcomes (*P* < 0.1); and clinical relevance. Variables that were highly correlated but clinically distinct were retained. To formally assess multicollinearity, variance inflation factor (VIF) was calculated for the final set of variables; all VIF values were <2, indicating no significant collinearity concerns. To account for within-patient clustering of vessels, generalized estimating equations (GEE) were used for subsequent analyses. To identify independent predictors of different ischemia phenotypes, separate GEE models were constructed with CFR+, FFR-CT+, and the four composite subgroups as dependent variables. Independent predictors were identified via univariate and multivariate logistic regression. For multiple comparisons, the Bonferroni correction was applied, with an adjusted *P* < 0.05 considered statistically significant. Two prediction models were developed using the same set of selected coronary features for CFR-defined and FFR-CT-defined ischemia. Discrimination was evaluated using the area under the receiver operating characteristic (ROC) curve (AUC), internally validated via 1,000 bootstrap iterations. Calibration was assessed using calibration curves and related metrics. A two-tailed *p*-value < 0.05 was considered statistically significant.

## 3 Results

### 3.1 Agreement analysis between CZT-SPECT CFR and FFR-CT

Of the 753 vessels analyzed, 262 were CFR+ and 491 were CFR-, while 122 were FFR-CT+ and 631 were FFR-CT−. Agreement analysis yielded a Kappa value of 0.084 (*P* = 0.009). Baseline clinical characteristics for these 251 patients are shown in Table 1.

**Table 1:** Baseline clinical characteristics.

Patient characteristics	Overall (*n* = 251)	Medication use	Overall (*n* = 251)
	
Age, years	60 (51, 68)	Anti-platelet, *n* (%)	182 (72.5%)
Male sex, *n* (%)	144 (57.4%)	Statins, *n* (%)	102 (40.6%)
BMI, kg/m2	25.61 ± 3.46	Beta-blockers, *n* (%)	129 (51.4%)
Risk factors		CCB, *n* (%)	108 (43.0%)
Hypertension, *n* (%)	135 (53.8%)	ACEI or ARB, *n* (%)	96 (38.2%)
Diabetes, *n* (%)	47 (18.7%)	Nitrate, *n* (%)	89 (35.5%)
Hyperlipidemia, *n* (%)	70 (27.9%)	Coronary characteristics	
Smoking history, *n* (%)	80 (31.9%)	Non-obstruction, *n* (%)	101 (40.2%)
Family history, *n* (%)	34 (13.5%)	Obstruction, *n* (%)	150 (59.8%)
LVEF (%)	62 (54, 65)	Typical angina, *n* (%)	83(33.1%)

BMI, body mass index; LVEF, left ventricular ejection fraction; CCB, calcium channel blocker; ACEI, angiotensin-converting enzyme inhibitor; ARB, angiotensin receptor blocker; Obstruction defined as ≥50% diameter stenosis in any major epicardial vessel assessed by coronary CT angiography.

To assess the robustness of our findings to the choice of CFR cutoff, we performed a sensitivity analysis using an alternative definition of impaired flow reserve (CFR <2.5). Under this definition, the prevalence of CFR+ at the vessel level was 52.1% (392/753). The agreement between CFR and FFR-CT remained poor, with a Kappa value of 0.080 (*P* = 0.002).

### 3.2 Univariate and multivariate logistic regression analyses across groups

**Figure 3:**
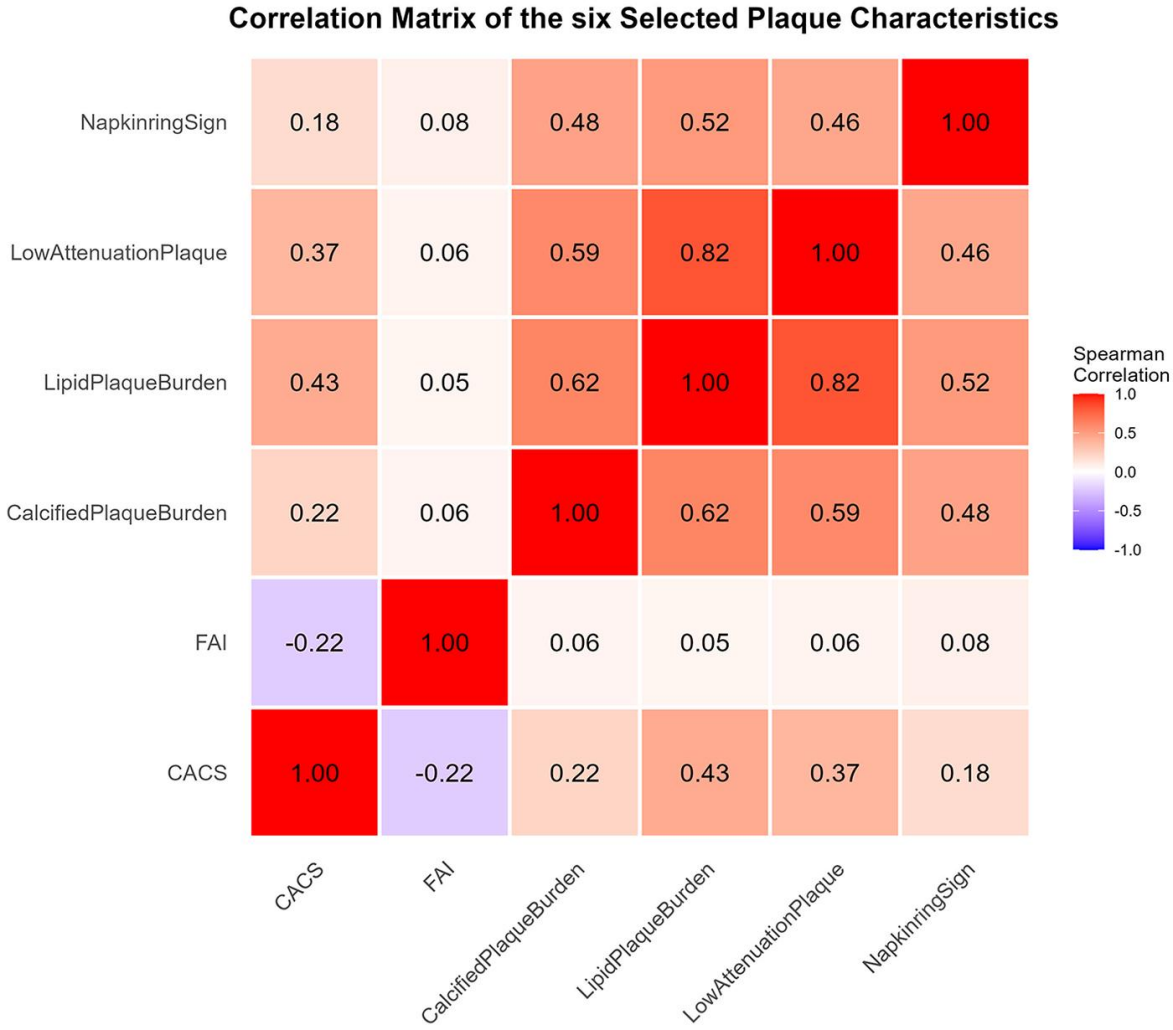
Spearman correlation analysis of CCTA-derived coronary features. CACS, coronary artery calcium scores; FAI, Perivascular fat attenuation index.

Through the selection process, six representative coronary parameters were retained for further analysis: CACS, FAI, calcified plaque burden, lipid plaque burden, napkin-ring sign, and low attenuation plaque. Details are shown in Figure 3.

Under the GEE model, for ischemia-defined vessels by CFR (CFR+), both FAI (OR: 1.035; 95% CI: 1.010–1.062; *P* = 0.007) and calcified plaque burden (OR: 1.032; 95% CI: 1.006–1.059; *P* = 0.015) were independent predictors in multivariate analysis. For ischemia-defined vessels by FFR-CT (FFR-CT+), FAI, calcified plaque burden, lipid plaque burden, and low attenuation plaque were all independent predictors (all *P* < 0.05), with low attenuation plaque showing the strongest association (OR: 3.527; 95% CI: 1.716–7.247; *P* < 0.001) in Table 2.

**Table 2:** Logistic regression analysis of abnormal CFR and FFR-CT.

Type	Variable	Univariate		Multivariate	
		OR (95%CI)	*P* value	OR (95%CI)	*P* value
CFR+ (*n* = 262)	CACS	0.999 (0.999–1.000)	**0** **.** **019**	0.999 (0.999–1.000)	0.112
	FAI	1.038 (1.014–1.063)	**0**.**002**	1.035 (1.010–1.062)	**0**.**007**
	CPB	1.032 (1.004–1.060)	**0**.**023**	1.032 (1.006–1.059)	**0**.**015**
	LPB	1.102 (0.993–1.222)	0.067	–	–
	NRS	1.069 (0.702–1.628)	0.756	–	–
	LAP	0.924 (0.669–1.276)	0.631	–	–
FFR-CT+ (*n* = 122)	CACS	1.001 (1.001–1.002)	**<0**.**001**	1.000 (0.999–1.001)	0.600
	FAI	1.044 (1.016–1.073)	**0**.**002**	1.067 (1.026–1.109)	**0**.**001**
	CPB	1.190 (1.146–1.236)	**<0**.**001**	1.183 (1.129–1.240)	**<0**.**001**
	LPB	1.961 (1.617–2.378)	**<0**.**001**	1.650 (1.335–2.041)	**<0**.**001**
	NRS	5.964 (3.881–9.166)	**<0**.**001**	1.367 (0.777–2.403)	0.278
	LAP	12.357 (6.489–23.534)	**<0**.**001**	3.527 (1.716–7.247)	**<0**.**001**

Bold type indicates *P* < 0.05; “–” indicates that the variable was not included in the multivariate model because *P* ≥ 0.05 in the univariate analysis. CFR, coronary flow reserve; FFR-CT, fractional flow reserve derived from coronary CT angiography; CACS, coronary artery calcium scores; FAI, perivascular fat attenuation index; CPB, calcified plaque burden; LPB, lipid plaque burden; NRS, napkin-ring sign; LAP, low attenuation plaque.

In the subgroup analysis, the CFR+/FFR-CT− group showed no independent associations with any features (all *P* > 0.05). In the CFR-/FFR-CT+ group, independent predictors included the napkin-ring sign (OR: 1.921; 95%CI: 1.028–3.591; *P* = 0.041) and low attenuation plaque (OR: 9.899; 95%CI: 2.899–34.122; *P* < 0.001). The CFR+/FFR-CT+ group had multiple independent predictors: FAI, calcified plaque burden, lipid plaque burden, and low attenuation plaque (all *P* < 0.05). For the CFR-/FFR-CT− group, FAI, calcified plaque burden, and lipid plaque burden were independent predictors (all *P* < 0.05) in Table 3.

**Table 3:** Logistic regression analysis of CFR combined with FFR-CT subgroups.

Type	Variable	Univariate		Multivariate	
		OR (95%CI)	*P* value	OR (95%CI)	*P* value
CFR+/FFR-CT− (*n* = 207)	CACS	0.999 (0.998–1.000)	**0** **.** **006**	0.999 (0.999–1.000)	0.072
	FAI	1.020 (0.996–1.044)	0.105	–	–
	CPB	0.970 (0.942–0.999)	**0**.**045**	0.986 (0.958–1.015)	0.343
	LPB	0.774 (0.648–0.925)	**0**.**005**	0.889 (0.770–1.026)	0.108
	NRS	0.573 (0.367–0.897)	**0**.**015**	0.836 (0.500–1.396)	0.493
	LAP	0.565 (0.405–0.789)	**<0**.**001**	0.786 (0.534–1.158)	0.223
CFR-/FFR-CT+ (*n* = 67)	CACS	1.002 (1.001–1.002)	**<0**.**001**	1.001 (1.000–1.002)	0.134
	FAI	1.015 (0.982–1.048)	0.381	–	–
	CPB	1.079 (1.002–1.162)	**0**.**045**	1.045 (0.977–1.119)	0.199
	LPB	1.304 (1.081–1.573)	**0**.**005**	1.081 (0.929–1.257)	0.314
	NRS	5.481 (3.236–9.286)	**<0**.**001**	1.921 (1.028–3.591)	**0**.**041**
	LAP	20.720 (6.421–66.867)	**<0**.**001**	9.899 (2.899–34.122)	**<0**.**001**
CFR+/FFR-CT+ (*n* = 55)	CACS	1.001 (1.000–1.001)	0.131	–	–
	FAI	1.074 (1.030–1.121)	**<0**.**001**	1.088 (1.027–1.153)	**0**.**004**
	CPB	1.100 (1.057–1.144)	**<0**.**001**	1.088 (1.049–1.129)	**<0**.**001**
	LPB	1.524 (1.265–1.835)	**<0**.**001**	1.342 (1.093–1.648)	**0**.**005**
	NRS	4.110 (2.202–7.670)	**<0**.**001**	1.150 (0.546–2.422)	0.714
	LAP	6.292 (2.995–13.217)	**<0**.**001**	2.456 (1.093–5.520)	**0**.**030**
CFR-/FFR-CT– (*n* = 424)	CACS	1.000 (0.999–1.001)	0.840	–	–
	FAI	0.962 (0.941–0.983)	**<0**.**001**	0.960 (0.938–0.982)	**<0**.**001**
	CPB	0.885 (0.850–0.920)	**<0**.**001**	0.884 (0.847–0.922)	**<0**.**001**
	LPB	0.705 (0.598–0.830)	**<0**.**001**	0.730 (0.607–0.879)	**<0**.**001**
	NRS	0.472 (0.325–0.684)	**0**.**004**	1.008 (0.655–1.551)	0.972
	LAP	0.590 (0.432–0.806)	**<0**.**001**	1.286(0.879–1.882)	0.195

Bold type indicates *P* < 0.05; “–” indicates that the variable was not included in the multivariate model because *P* ≥ 0.05 in the univariate analysis. CFR, coronary flow reserve; FFR-CT, fractional flow reserve derived from coronary CT angiography; CACS, coronary artery calcium scores; FAI, perivascular fat attenuation index; CPB, calcified plaque burden; LPB, lipid plaque burden; NRS, napkin-ring sign; LAP, low attenuation plaque.

### 3.3 Comparison among subgroups combining CFR and FFR-CT

For the six representative features, multiple comparisons were performed using the Bonferroni method within the GEE model. Comparison of CACS revealed that the CFR+/FFR-CT- group was significantly lower than both FFR-CT abnormal groups (vs. CFR-/FFR-CT+, adjusted *P* < 0.001; vs. CFR+/FFR-CT+, adjusted *P* = 0.034). Additionally, the CFR-/FFR-CT+ group had a significantly higher CACS than the CFR-/FFR-CT- group (adjusted *P* = 0.012). For FAI, only the CFR+/FFR-CT+ group [−75.36 (−79.27, −70.85) HU] was significantly higher than the CFR-/FFR-CT- group [−79.99 (−85.52, −74.89) HU] (adjusted *P* < 0.001).

Regarding calcified plaque burden, lipid plaque burden, napkin-ring sign, and low attenuation plaque, both the CFR-/FFR-CT+ and CFR+/FFR-CT+ groups showed significantly higher values than the CFR+/FFR-CT− and CFR-/FFR-CT- groups (all adjusted *P* < 0.05), while no significant differences were observed between the former two or between the latter two groups in Table 4.

**Table 4:** Comparisons between CFR combined with FFR-CT subgroups.

Variable	CFR+/FFR-CT− (*n* = 207)	CFR-/FFR-CT + (*n* = 67)	CFR+/FFR-CT + (*n* = 55)	CFR-/FFR-CT− (*n* = 424)
CACS	64.16 (0.08, 229.66) ^a^	236.83 (155.14, 413.94) ^b^	224.38 (35.20, 338.92) ^b,c^	136.76 (1.69, 312.05) ^a,c^
FAI	−77.20(−83.48, −72.35) ^a,b^	−77.39(−82.75, −73.63) ^a,b^	−75.36(−79.27, −70.85) ^a^	−79.99(−85.52, −74.89) ^b^
CPB	0.13 (0.00, 2.90) ^a^	7.09 (2.24, 14.73) ^b^	5.15 (1.83, 16.37) ^b^	0.07 (0.00, 1.80) ^a^
LPB	0.00 (0.00, 0.37) ^a^	1.10 (0.35, 2.02) ^b^	1.05 (0.27, 3.35) ^b^	0.01 (0.00, 0.42) ^a^
NRS	35 (16.9%) ^a^	39 (58.2%) ^b^	29 (52.7%) ^b^	75 (17.7%) ^a^
LAP	92 (44.4%) ^a^	64 (95.5%) ^b^	48 (87.3%) ^b^	208(49.1%) ^a^

Different superscript letters (^a,b,c^) indicate statistically significant differences between groups in pairwise comparisons based on the Bonferroni correction (adjusted *P* < 0.05), while groups sharing at least one same letter do not differ significantly. CFR, coronary flow reserve; FFR-CT, fractional flow reserve derived from coronary CT angiography; CACS, coronary artery calcium scores; FAI, perivascular fat attenuation index; CPB, calcified plaque burden; LPB, lipid plaque burden; NRS, napkin-ring sign; LAP, low attenuation plaque.

### 3.4 Predictive performance of features for CFR and FFR-CT

**Figure 4:**
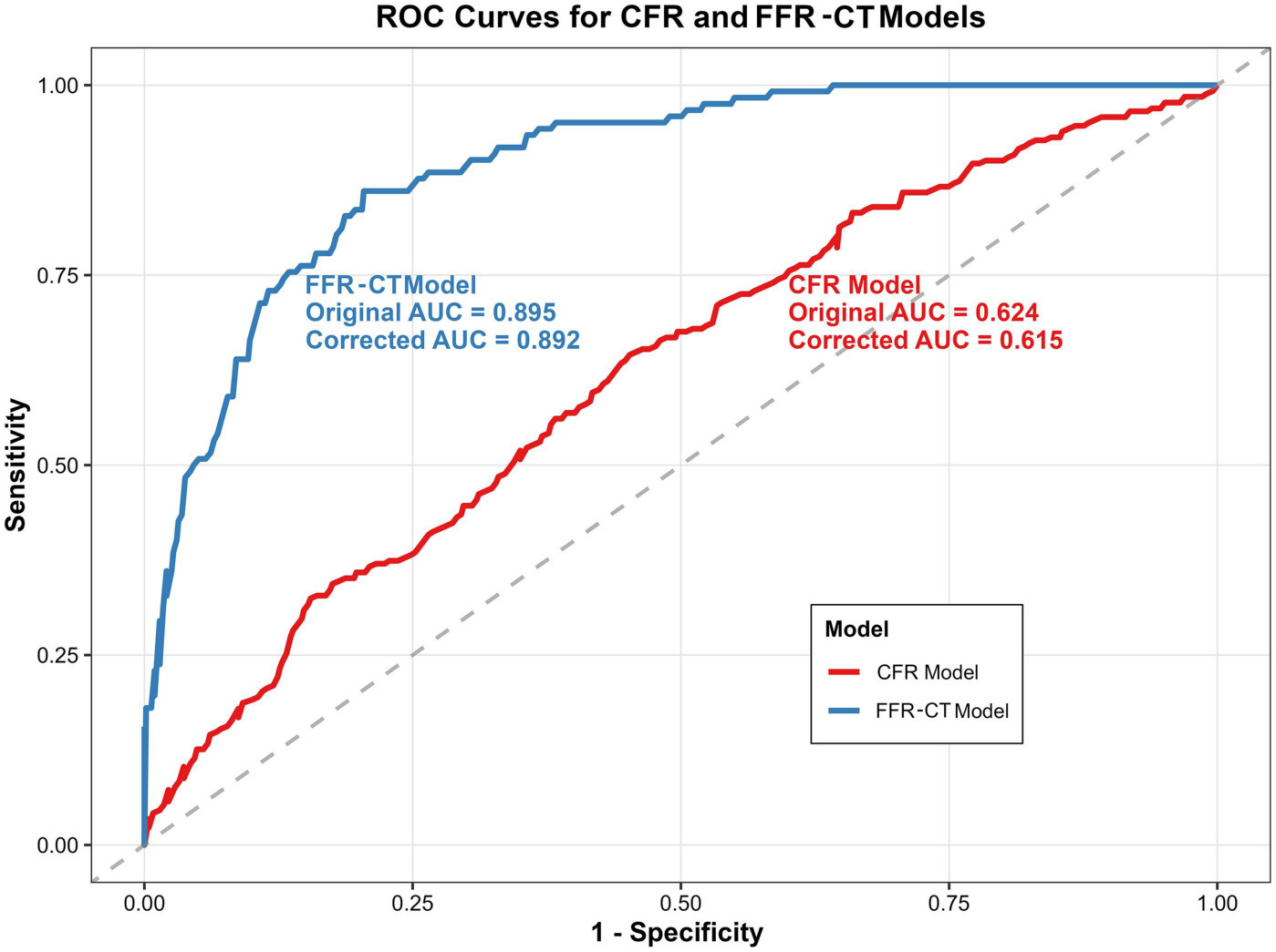
ROC curves for predicting CFR and FFR-CT using the same Set of parameters. CFR, coronary flow reserve; FFR-CT, fractional flow reserve derived from coronary CT angiography.

The predictive performance of the six plaque features was evaluated for each outcome. For discrimination (Figure 4), the bootstrapped internal-validated AUC was 0.892 (0.886–0.896) for the FFR-CT model and 0.615 (0.600–0.623) for the CFR model. The optimism was 0.3% and 0.9%, respectively, indicating good fit.

**Figure 5:**
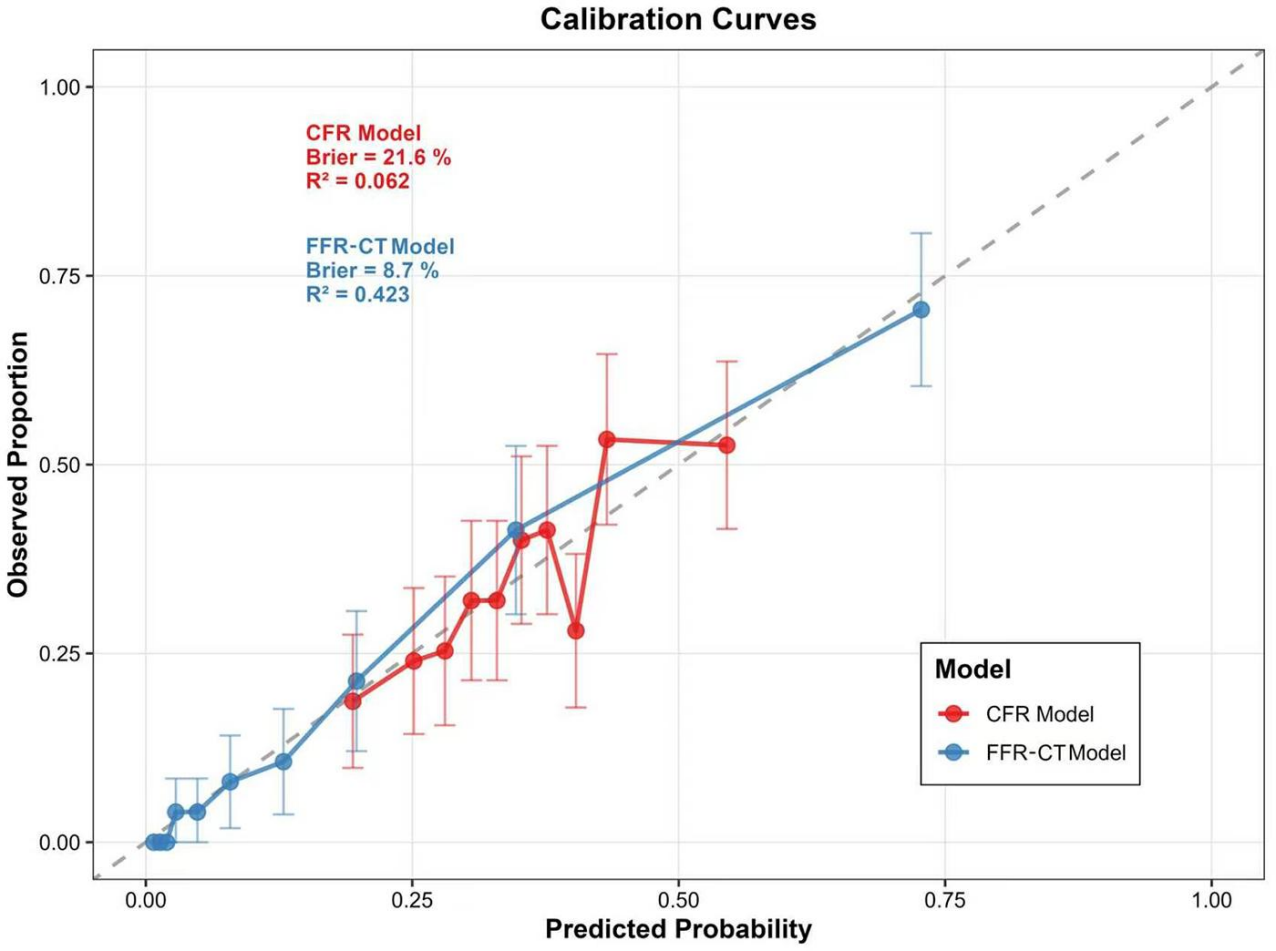
Calibration curves of the Two prediction models. CFR, coronary flow reserve; FFR-CT, fractional flow reserve derived from coronary CT angiography.

Regarding calibration, the Brier Score was 8.7% for the FFR-CT model and 21.6% for the CFR model; both models demonstrated acceptable calibration (Hosmer-Lemeshow *P* > 0.05). See Table 5 and Figure 5 for details.

**Table 5:** Calibration performance of the predictive model for CFR and FFR-CT.

Metric	CFR	FFR-CT
Somers’ Dxy	0.248	0.791
R^2(^Nagelkerke)	0.062	0.423
Brier Score	21.6%	8.7%
Calibration Slope	1	1
Intercept	0	0
E-max	0.124	0.066
Hosmer-Lemeshow X^2^	7.7	6.6
Hosmer-Lemeshow *P* value	0.460	0.578

CFR, coronary flow reserve; FFR-CT, fractional flow reserve derived from coronary CT angiography. *Somers’ Dxy*: rank correlation between predicted probabilities and observed outcomes. *R² (Nagelkerke)*: proportion of variance explained by the model. *Brier Score*: mean squared difference between predicted probabilities and observed outcomes. *E-max*: maximum absolute difference between predicted and observed probabilities. *Hosmer-Lemeshow χ² and P*: goodness-of-fit test; *P* > 0.05 indicates acceptable calibration.

### 3.5 Sensitivity analysis excluding RCA vessels

To address potential heterogeneity in FAI measurement across vessels, we performed a sensitivity analysis restricted to LAD and LCX vessels (*n* = 502), in which FAI was measured using a uniform proximal 40 mm segment. In this subgroup, the agreement between CFR and FFR-CT remained poor (Kappa = 0.047, *P* = 0.242). Multivariable GEE analysis showed that for CFR+, FAI (OR: 1.037, 95% CI: 1.008–1.067, *P* = 0.013) and calcified plaque burden (OR: 1.042, 95% CI: 1.013–1.071, *P* = 0.004) remained independent predictors, while CACS showed a negative association (OR: 0.998, 95% CI: 0.997–0.999, *P* = 0.003). For FFR-CT+, CACS, lipid and calcified plaque burden, high-risk plaque features including low attenuation plaque and napkin-ring sign remained independent predictors (all *P* < 0.05), whereas FAI was no longer significant. See Supplementary Table S1.

## 4 Discussion

This study explored the pathophysiological mechanisms underlying the discordance between CFR and FFR-CT at the vessel level by integrating AI-derived CCTA-based coronary features with two distinct functional ischemia markers. The results revealed a clear asymmetric relationship between the two ischemia indicators: reduced CFR was associated with features of vascular inflammation and diffuse burden (such as FAI and calcified plaque burden), whereas decreased FFR-CT was more closely associated with high-risk plaque characteristics leading to luminal stenosis (e.g., low attenuation plaque). Further analysis showed that vessels with discordant results exhibited distinct plaque phenotypes, the same set of coronary features achieved an adjusted AUC of 0.892 for FFR-CT-defined ischemia and 0.615 for CFR-defined ischemia. Together, these findings indicate that CFR and FFR-CT reflect distinct yet complementary pathophysiological dimensions of myocardial ischemia. However, because this study lacked invasive reference standards—specifically, invasive fractional flow reserve (FFR) for epicardial stenosis (21) and the index of microcirculatory resistance (IMR) for microvascular function (22)—our findings should be interpreted as describing associations between plaque characteristics and non-invasive test results, rather than establishing the superiority or diagnostic accuracy of either modality.

In this study, the agreement analysis between CFR and FFR-CT showed a Kappa value of only 0.084 (*P* = 0.009), indicating very poor consistency in their assessment of myocardial ischemia. This finding aligns with the distinct physiological functions reflected by CFR, which represents overall flow reserve from epicardial arteries to the microcirculation, and FFR-CT, which estimates the pressure gradient across epicardial stenoses, and is consistent with prior research (13, 23).

It is worth noting that the optimal threshold for defining impaired CFR using CZT-SPECT remains an area of ongoing investigation. Thresholds ranging from 2.0 to 2.5 have been employed across different modalities and study populations, reflecting variability in tracer kinetics, acquisition protocols, and reference standards. In the present study, we selected a threshold of <2.0 based on prior validation studies demonstrating its prognostic utility in CZT-SPECT cohorts (9, 24, 25). To assess the potential impact of threshold selection on our conclusions, we performed a sensitivity analysis using an alternative cutoff of <2.5. While this higher threshold increased the prevalence of CFR+ from 34.8% to 52.1%, the poor agreement between CFR and FFR-CT persisted (Kappa = 0.080 vs. 0.084). This sensitivity analysis supports the robustness of our core finding that CFR and FFR-CT capture fundamentally distinct aspects of coronary pathophysiology, independent of the specific threshold used to define CFR impairment. Nevertheless, it is undeniable that the classification of CFR+ is threshold-dependent, and the absolute prevalence estimates may vary across studies using different definitions.

Considering the multicollinearity among coronary characteristic parameters, this study initially filtered irrelevant variables through correlation analysis and univariate screening. Guided by clinical knowledge, we ultimately selected six coronary characteristic parameters representing: overall calcification burden (CACS), inflammation and activity (FAI), plaque composition (calcified and lipid components), and plaque vulnerability (napkin-ring sign and low attenuation plaque). In the GEE model, logistic regression analyses were conducted for the selected feature parameters under different ischemic backgrounds. Under the CFR+ condition, multivariate analysis showed that only FAI and calcified plaque burden were independent predictors. Studies have shown (26–28) that inflammatory mediators released by the diseased vessel wall can diffuse through paracrine signaling to perivascular adipose tissue, affecting lipid accumulation and thereby altering the lipid content around the lesion. FAI can capture this change and subsequently reflect perivascular inflammatory activity, which is closely associated with endothelial dysfunction and microcirculatory impairment. Moreover, calcified plaque burden is calculated as the volume of calcified plaque divided by the volume of the lesion vessel. Extensive calcified burden may indicate a more advanced stable plaque state (29), reflecting the chronic and overall load of atherosclerosis. Such diffuse lesions are often accompanied by widespread endothelial dysfunction and increased microvascular resistance. Therefore, this suggests that reduced CFR may be more closely related to diffuse vascular inflammation, microcirculatory dysfunction, and overall atherosclerotic burden, rather than merely vascular stenosis or vulnerable plaques. In the context of FFR-CT+, independent predictors include multiple coronary characteristic parameters, with low attenuation plaque being the most relevant. Currently, high-risk plaques are clinically significant and robust markers of vulnerable, rupture-prone lesions (30). Among these, low attenuation plaques represent necrotic cores rich in lipids, which are not only strongly associated with MACE (31) but also constitute a key pathological factor leading to luminal stenosis, hemodynamic impairment, and ultimately ischemic lesions (32). Additionally, lipid plaque burden represents the total volume of “soft plaques” causing luminal narrowing, reflecting plaque characteristics responsible for local flow obstruction (33). Furthermore, FAI and calcified plaque burden also serve as independent predictors in this context, indicating that inflammation and overall atherosclerotic burden are contributing factors to hemodynamic impairment. This suggests that a reduced FFR-CT may be more closely associated with luminal stenosis and the development of flow-limiting lesions.

Additionally, this study further classified vessels into four subgroups based on the presence or absence of CFR and FFR-CT reduction. Regression analysis showed that for the CFR+/FFR-CT- group, no independent predictors were identified among the six plaque characteristics examined. This finding suggests that plaque features alone are insufficient to explain the presence of ischemia in this subgroup, raising the hypothesis that non-plaque factors—such as coronary microvascular resistance, heart rate, or myocardial factors—may play a predominant role (34, 35). However, direct confirmation of this hypothesis requires dedicated microvascular function assessment (such as IMR), which was not available in the present study. For the CFR-/FFR-CT+ group, the napkin-ring sign and low attenuation plaque were identified as independent predictors, indicating that this vessel type is associated with stenotic lesions caused by high-risk, vulnerable focal plaques (36). For the CFR+/FFR-CT+ group, it is noteworthy that its independent predictors were the same as those in the FFR-CT+ context. We hypothesize that the FFR-CT+ cohort resembles a broad population representing all vessels with hemodynamically significant stenosis. Within this population, one subset (CFR-/FFR-CT+) exhibits only stenosis with preserved microcirculatory function, while the other subset (CFR+/FFR-CT+) demonstrates both stenosis and superimposed microcirculatory dysfunction. The high-risk plaque features identified in CFR-/FFR-CT+ may represent necessary conditions for initiating the ischemic cascade. When further combined with the inflammatory state and high atherosclerotic load reflected by FAI and calcified plaque burden, a more severe systemic or diffuse pathological state (CFR+/FFR-CT+) ensues. This suggests that CFR+/FFR-CT+ represents an aggravated stage of CFR-/FFR-CT+, sharing the same underlying plaque anatomy but differing in severity and extent of involvement. For the CFR-/FFR-CT− group, independent predictors included FAI, calcified plaque burden, and lipid plaque burden, yet all exhibited OR <1. This indicates that the presence of these features actually reduced the likelihood of a vessel being classified into this group, providing inverse evidence that their existence is regarded as an abnormal state.

To further delineate the coronary characteristic profiles across these groups, this study also compared coronary feature parameters among the four subgroups. The results showed that CACS was higher in groups with abnormal FFR-CT, suggesting that total calcified burden is associated with plaque accumulation that leads to stenosis (37). In contrast, the low CACS in the CFR+/FFR-CT− group reaffirms that ischemia indicated by reduced CFR is more likely driven by factors beyond traditional plaque burden (34). FAI was only significantly higher in the CFR+/FFR-CT+ group compared with the CFR-/FFR-CT− group, reaffirming that inflammation serves as a bridge linking stenosis with diffuse microvascular dysfunction. When inflammation coexists with stenosis, it may be more likely to induce a comprehensive pathological state (33, 38). Taken together, these observations highlight the complementary nature of CACS and FAI in CAD. CACS reflects cumulative atherosclerotic burden and aligns more closely with structural stenosis (FFR-CT), whereas FAI captures active vascular inflammation and is more intimately linked to microvascular dysfunction (CFR). Rather than competing markers, they provide distinct yet synergistic information—one representing historical disease load, the other current inflammatory activity. This conceptual framework, recently articulated by Savo et al., supports the integration of both markers for more comprehensive phenotyping of ischemic risk (39). Regarding other parameters, including high-risk plaque features, both FFR-CT abnormal groups showed significantly higher values than the two FFR-CT normal groups. This further validates that these features are primarily associated with flow-limiting stenosis and are relatively independent of whether CFR is reduced.

Finally, using a fixed set of plaque features, we evaluated their predictive performance for ischemia as defined by two different physiological standards: CFR <2.0 and FFR-CT ≤0.8. The FFR-CT model achieved an adjusted AUC of 0.892 with a Brier score of 8.7%, and the CFR model achieved an AUC of 0.615 with a Brier score of 21.6%. These findings suggest that AI-derived anatomical plaque features from CCTA are more closely associated with obstructive ischemia as defined by FFR-CT, whereas their association with CFR-defined ischemia is limited. Several factors may explain this disparity. First, FFR-CT and plaque features are derived from the same CCTA dataset, which may inflate their apparent association. Second, and more fundamentally, there exists an inherent mismatch between the level of analysis and the nature of the measurement: plaque characteristics were quantified at the vessel level (the epicardial coronary artery), whereas CFR derived from CZT-SPECT represents a territory-level assessment of microvascular function, integrating perfusion over the entire myocardial territory supplied by that vessel. This mismatch may introduce misclassification, as a vessel-level plaque feature cannot fully capture territory-level microvascular function, which is also influenced by distal microvascular health, myocardial factors, and collateral circulation (34). While the GEE model accounts for within-patient clustering, this limitation should be considered when interpreting the CFR-related findings. Future studies employing vessel-specific CFR measurements (e.g., invasive Doppler/thermodilution) would be better suited for vessel-level analyses.

Since the measurement protocol for FAI differed by vessel, we performed a sensitivity analysis restricted to LAD and LCX vessels to assess whether this heterogeneity affected our findings. The results were partially consistent with the primary analysis. For CFR <2.0, FAI and calcified plaque burden remained significant predictors, consistent with the main analysis. The emergence of CACS as an additional predictor with an odds ratio <1 likely reflects statistical competition with calcified plaque burden, given their high collinearity. For FFR-CT ≤0.8, CACS, lipid and calcified plaque burden, high-risk plaque features including low attenuation plaque and napkin-ring sign remained independent predictors, corroborating the primary finding that these features are closely linked to obstructive ischemia. However, FAI did not retain independent predictive value in this subgroup, which may be attributable to reduced statistical power (the number of FFR-CT+ vessels decreased from 122 to 93) or to genuine differences in the inflammatory contribution of RCA vs. LAD/LCX vessels. Overall, this sensitivity analysis supports the robustness of our core conclusions, while highlighting the importance of methodological standardization in FAI measurement across different coronary vessels.

This study has several limitations. First, it is a single-center retrospective study with a relatively small sample size. Second, a significant limitation of this study is the absence of invasive reference standards. Consequently, we cannot determine which of the two non-invasive modalities more accurately reflects true physiological ischemia. Future studies incorporating invasive reference standards are needed to validate the comparative accuracy of these modalities and to determine their respective roles in guiding clinical decision-making. Third, the analysis focused on coronary plaque characteristics and did not adequately incorporate patient-level clinical factors. Fourth, a fundamental limitation of this study is that both the CCTA coronary features and FFR-CT are derived from the same imaging dataset. While FFR-CT is a computational simulation of coronary flow based on anatomical geometry, and coronary features are direct anatomical measurements, their strong association (AUC = 0.892) is at least partially attributable to the shared data source. This precludes a truly independent validation of the predictive model for FFR-CT. Future external validation cohorts are needed to confirm the generalizability of these findings.

In summary, this study systematically explored the associations and distinctions between AI-derived CCTA coronary features and two distinct myocardial ischemia criteria—CFR <2.0 and FFR-CT ≤0.8—at the vessel level. Our findings indicate that CFR <2.0 is more associated with inflammation and diffuse atherosclerotic burden, whereas FFR-CT ≤0.8 reflects focal, obstructive ischemia driven by high-risk plaques such as low attenuation plaque. Although CCTA remains a cornerstone for assessing obstructive coronary lesions, its morphological features alone are insufficient for predicting abnormal CFR, which is more closely linked to coronary microvascular function. These findings provide a framework for integrating complementary physiological perspectives to enable more precise phenotyping of CAD. However, given the absence of invasive validation, the results should be considered hypothesis-generating and require confirmation in future studies incorporating invasive physiological assessment.

## Data Availability

The raw data supporting the conclusions of this article will be made available by the authors, without undue reservation.
