# Supplementary material for: Associations of AI-derived coronary CT angiography features with CZT-SPECT coronary flow reserve and FFR-CT
Source: Front Cardiovasc Med. 2026 Apr 21;13:1803774. doi: 10.3389/fcvm.2026.1803774 (PMC13138943; doi:10.3389/fcvm.2026.1803774)
Supplement: Supplementary file 1 [file Table1.doc]

**Supplementary Table S1. Sensitivity analysis excluding RCA vessels**

| Type | Variable | Univariate | | Multivariate | |
| --- | --- | --- | --- | --- | --- |
|  |  | OR (95%CI) | P value | OR (95%CI) | P value |
| CFR+  (n=184) | CACS | 0.998(0.997–0.999) | **<0.001** | 0.998(0.997–0.999) | **0.003** |
|  | FAI | 1.042(1.015–1.069) | **0.002** | 1.037(1.008–1.067) | **0.013** |
|  | CPB | 1.029(1.002–1.058) | **0.036** | 1.042(1.013–1.071) | **0.004** |
|  | LPB | 0.953(0.806–1.128) | 0.579 | **-** | **-** |
|  | NRS | 1.113(0.703–1.764) | 0.647 | **-** | **-** |
|  | LAP | 0.909(0.643–1.287) | 0.591 | **-** | **-** |
| FFR-CT +(n=93) | CACS | 1.006(1.004–1.007) | **<0.001** | 1.003(1.001–1.005) | **0.002** |
|  | FAI | 1.025(0.994–1.057) | 0.115 | - | **-** |
|  | CPB | 1.185(1.134–1.240) | **<0.001** | 1.163(1.102–1.226) | **<0.001** |
|  | LPB | 2.043(1.511–2.763) | **<0.001** | 1.496(1.180–1.898) | **<0.001** |
|  | NRS | 7.559(4.607–12.402) | **<0.001** | 2.172(1.201–3.930) | **0.010** |
|  | LAP | 11.874(6.001–23.497) | **<0.001** | 2.708(1.220–6.011) | **0.014** |

Bold type indicates P < 0.05; "-" indicates that the variable was not included in the multivariate model because P ≥ 0.05 in the univariate analysis. CFR: coronary flow reserve; FFR-CT: fractional flow reserve derived from coronary CT angiography; CACS: coronary artery calcium scores; FAI: perivascular fat attenuation index; CPB: calcified plaque burden; LPB: lipid plaque burden; NRS: napkin-ring sign; LAP: low attenuation plaque.
